# Healthcare resource utilization and costs in 23–25-year-old women with human papillomavirus (HPV) associated anogenital diseases in Germany – a retrospective analysis of statutory health insurance claims data

**DOI:** 10.1186/s12913-022-08397-1

**Published:** 2022-08-05

**Authors:** Miriam Reuschenbach, Sarah Mihm, Regine Wölle, Kim Maren Schneider, Christian Jacob, Wolfgang Greiner, Monika Hampl, Elizabeth Goodman

**Affiliations:** 1 Global Medical and Scientific Affairs, MSD Sharp and Dohme GmbH, Munich, Germany; 2 Department of Market Access, MSD Sharp and Dohme GmbH, Munich, Germany; 3 EU Real World Evidence, Xcenda GmbH, Hannover, Germany; 4 Department of Health Economics and Health Care Management, Bielefeld School of Public Health, Bielefeld University, Bielefeld, Germany; 5 Department of Obstetrics and Gynecology, University Hospital of Düsseldorf, Düsseldorf, Germany; 6 Center for Observational and Real-World Evidence (CORE), Merck and Co., Inc, Rahway, NJ USA

**Keywords:** HPV, Anogenital diseases, Healthcare costs, Healthcare resource utilization, Women, Claims data, Germany

## Abstract

**Background:**

Human papilloma virus (HPV) causes multiple anogenital diseases including cervical cancer and is the most common sexually transmitted infection. Healthcare resource utilization (HRU) associated with HPV-related anogenital diseases includes diagnostic and disease specific treatment regimens. A recent study showed disease burden of young women aged 23–25 years, who were the first populations eligible to receive HPV vaccination after its introduction in Germany. Cost for the German statutory health insurance (SHI) due to HPV‑related anogenital diseases in this population are unknown. This study aimed at assessing HRU and costs related to HPV-associated anogenital diseases for the Germany SHI.

**Methods:**

We used a retrospective, matched cohort design to leverage the prior identified cohort of 23–25-year-old women born between 1989–1992 diagnosed with HPV-related anogenital disease from the Institute for Applied Health Research Berlin (InGef) Research Database. German SHI claims data from 2012–2017 were analyzed. The prior identified cases were matched (direct, without replacement) to women without anogenital diseases (1:10 ratio). HRU and costs for inpatient care, outpatient care, and pharmaceutical during a 3-year observation period were determined for both cases and controls and increments between the groups were assessed.

**Results:**

2,972 women diagnosed with anogenital diseases (cases) who were matched to 29,720 women without anogenital diseases (controls). Cases had more outpatient visits (52.4 visits vs. 39.2 visits) and more cases (45.2% vs. 31.7%) were hospitalized at least once in the 3‑year observation period. Most common outpatient procedures performed in cases were conization of the cervix uteri (4.4% cases; *n* < 5 controls), followed by other excision and destruction of diseased tissue of the cervix uteri (3.1% in cases; 0.0% in controls). Median difference in total healthcare costs of €684 (mean difference: €1,089, 95%CI: €752–1,426) suggest that HPV-related anogenital diseases were responsible for approximately €3.2 Million more healthcare costs for the identified cases in the four birth cohorts within the 3‑year observation period in the InGef Research Database. Costs were mainly driven by outpatient care (41.6% of total costs).

**Conclusion:**

In Germany, HPV-related anogenital diseases among young women are associated with considerable HRU and financial expenditures, mostly driven by outpatient care.

**Supplementary Information:**

The online version contains supplementary material available at 10.1186/s12913-022-08397-1.

## Background

Human Papillomavirus (HPV) is the most common sexually transmitted infection. Most sexually active individuals acquire HPV at least once in their lives [1]. In women, HPV infection may lead to several anogenital diseases, including genital warts as well as cervical, vulvar, vaginal, and anal precancer, as well as cancer of respective sites [1]. Although HPV can be acquired throughout life [2], the peak prevalence of cervical HPV infection in most European countries is before the age of 25 years [3]. HPV infections in young women are mostly transient and may clear within several months [4]. However, some persist, leading to HPV- associated diseases which create a significant burden to women and healthcare systems.

In a recent study we demonstrated a substantial burden of HPV-associated anogenital diseases in 23–25-year-old women in Germany between 2012–2017 based on statutory health insurance (SHI) claims data [5]. The identified women were of special interest as these 23–25-year-olds were from the first birth cohorts eligible for HPV vaccination as adolescents after its introduction in Germany (birth cohorts 1989–1992) and because their identification enabled exploration of the burden of HPV-associated anogenital diseases in these young, less well studied age groups. The most frequent diagnoses were anogenital warts with 3-year administrative prevalence rates for each birth cohort of 0.82–1.30% and grade 1 and grade 3 cervical intraepithelial neoplasia (CIN), with 3-year administrative prevalence rates of 1.31–1.60% for grade 1 lesions and 0.71–1.09% for grade 3 lesions [5].

The healthcare resources utilization and costs associated with the screening, diagnosis, and treatment of these HPV-related anogenital diseases in Germany is not well described, particularly for young women [6–8]. The few available studies mostly used medical records for cost estimates of selected anogenital diseases and focused on older patient groups. German women are recommended to see a gynecologist once a year and get an annual cytology test for cervical cancer screening once they reach the age of 20 years (in 2020 the guidelines changed to HPV/cytology co-testing every three years for those 35 and older) [9]. According to German guidelines, for genital warts, topical treatment may be applied using for example podophyllotoxin 0.5%, imiquimod 5%, or sinecatechine (green tea) 10% but also surgical techniques may be applied, like laser or electro-coagulation [10]. For grade 1 anogenital lesions it is recommended to carefully watch and wait [11, 12]. High-grade anogenital (cervical) lesions should be removed surgically, e.g., by conization. However, for women up to 24 years and depending on CIN grade, a conservative strategy should (CIN 2) or may (CIN 3) be applied, including regular examinations, colposcopy, cytology, and HPV testing [11].

As healthcare resource utilization for anogenital diseases and associated costs have been quantified incompletely, the actual healthcare expenditures for the German statutory health insurance (SHI) are mostly unknown, especially concerning young women. This study addresses this gap in the literature using the 23–25-year-old women born between 1989–1992 identified in our prior study of HPV-associated anogenital disease burden [5]. This follow-up study assesses the healthcare resource utilization and costs for the Germany SHI of these women over a 3-year period.

## Methods

### Study population

In this study, we used the study population of women in the Institute for Applied Health Research Berlin (InGef) Research Database from 2012–2017 who were born between 1989–1992 and who were identified for the previous work [5]. All women needed to be continuously observable from the age of 23–25, except for women who died during the study period. Identification of HPV-associated anogenital diseases was based on International Statistical Classification of Diseases, German Modification (ICD-10-GM) codes. Women with at least one documented ICD-10-GM diagnosis record for HPV-associated anogenital diseases in the outpatient sector (verified diagnoses) and in the inpatient sector (main or secondary diagnoses) were identified. In addition to the ICD-10-GM codes reported in our previous work, we also used dysplasia codes unspecified in terms of disease grade (see Table 1 for list of all included ICD-10-GM codes).

**Table 1:** List of ICD-10-GM codes for identification of HPV-associated anogenital diseases

Group	Description	ICD-10-GM Code
HPV infection	Papillom virus infection	B97.7
Warts	Anogenital (venereal) warts (condylomata)	A63.0
Grade 1	Other specified diseases of anus and rectum (AIN I & II)	K62.8
	Mild cervical dysplasia (CIN I)	N87.0
	Mild vaginal dysplasia (VaIN I)	N89.0
	Mild vulvar dysplasia (VIN I)	N90.0
Grade 2	Moderate cervical dysplasia (CIN II)	N87.1
	Moderate vaginal dysplasia (VaIN II)	N89.1
	Moderate vulvar dysplasia (VIN II)	N90.1
Grade 3	Carcinoma in situ of anus and anal canal (AIN III)	D01.3
	Carcinoma in situ of cervix uteri (CIN III)	D06.-
	Carcinoma in situ – Endocervix	D06.0
	Carcinoma in situ –Ectocervix	D06.1
	Carcinoma in situ of vulva (VIN III)	D07.1
	Carcinoma in situ of vagina (VaIN III)	D07.2
	Severe cervical dysplasia	N87.2
	Severe vaginal dysplasia, other	N89.2
	Severe vulvar dysplasia, other	N90.2
Carcinoma	Malignant neoplasm of cervix uteri	C53.-
	Malignant neoplasm of anus and anal canal	C21.-
	Malignant neoplasm of vulva	C51.-
	Malignant neoplasm of vagina	C52
Other	Dysplasia of cervix uteri	N87
	Dysplasia of cervix uteri, unspecified	N87.9
	Dysplasia of vagina, unspecified	N89.3
	Dysplasia of vulva, unspecified	N90.3

Abbreviations*: AIN* Anal intraepithelial neoplasia, *CIN* Cervical intraepithelial neoplasia*, HPV* Human papilloma virus, *ICD-10-GM* International Classification of Disease, 10th Revision, German Modification, *VaIN* Vaginal intraepithelial neoplasia, *VIN* Vulvar intraepithelial neoplasia

Women without a record of anogenital diseases as stated above during the 3-year observation period were determined eligible as matched controls. Controls were drawn at random from the pool of women without anogenital diseases. 23–25-year-old women with HPV-associated anogenital diseases and women without anogenital diseases were matched on a 1:10 ratio (direct matching, without replacement) based on age and gender and were compared in terms of healthcare costs and resource utilization during a 3-year observation period.

### Data source

For this study, we analyzed claims data from the SHI in Germany. Approximately 88% of the German population, corresponding to about 73 million individuals, is insured under the SHI [13, 14]. In 2020, the SHI was structured into approximately 97 separate and independent health insurances [15]. All individual health insurances offer the same comprehensive benefit package, as the contents to be reimbursed are set in Social Law and does not vary by region. Nearly full coverage for all healthcare related services is provided as only little co-payments exist. Therefore, the funds paid by the SHI to any provider of healthcare (e.g., hospital, physician, or pharmacist) represent almost the complete picture of total healthcare cost on an individual patient basis. All data may be linked to patient demographics including age, gender, and the type of occupation for individuals within the workforce.

The analysis were conducted using German SHI claims data from the “Institute for Applied Health Research Berlin” (InGef) research database. The InGef database consists of 8 million covered lives and includes the healthcare resource utilization and costs of services in an anonymized case-by-case individual format. For scientific research projects, an adjusted analysis sample of the InGef database has been created which includes approximately 4 million covered lives structured to represent the German population in terms of age and gender (structure of age and gender according to the Federal Office of Statistics (Statistisches Bundesamt, DESTATIS)) [16].

This InGef Research Database comprises healthcare claims data from about 60 different health insurances (corresponding to approximately two thirds of the overall number of health insurances in Germany). Furthermore, the sample represents ~ 4.8% of the German population and ~ 5.6% of the German SHI population. The InGef Research Database includes a well-distributed geographic representation of the population of Germany. Moreover, the InGef Research Database has proven to have good external validity to the German population in terms of morbidity, mortality, and drug utilization [17, 18].

### Data protection

By German legislation, the analysis of claims data from the SHI is permitted and does not require the approval of an ethics committee. Claims data from the participating SHIs are joined in a specialized trust center, anonymized, and transferred to the InGef afterwards. Due to data protection regulations the raw dataset is not allowed to leave the secured storage facilities, therefore, all analyses were conducted at InGef by an employee in accordance with a pre-specified data analysis plan.

### Outcomes

All outcomes were analyzed during the respective 3-year observation period for each birth cohort covering in sum the study period 2012–2017. Depending in the year of birth, the 3-year observation period covers different years of the complete study period (2012–2017). The following 3-year observation periods were considered for the analysis of study outcomes:

- Birth cohort 1989: 3-year observation period from 2012–2014
- Birth cohort 1990: 3-year observation period from 2013–2015
- Birth cohort 1991: 3-year observation period from 2014–2016
- Birth cohort 1992: 3-year observation period from 2015–2017

All results are reported for the complete study period 2012–2017.

#### Healthcare costs

To estimate the healthcare costs of HPV-associated anogenital diseases, all-cause costs during the observation period were assessed for cases as well as their matched controls. All healthcare costs (in Euro, without inflation adjustments) reimbursed by the SHI during the respective observation periods were assessed in total and stratified by healthcare domain (inpatient care, outpatient care, and pharmaceuticals).

#### Resource use

The resource utilization was assessed in terms of all outpatient physician visits, hospitalizations, performed outpatient/inpatient services and procedures, and pharmaceutical prescriptions, which were reimbursed by the SHI in the respective observation periods for both the cases as well as the matched controls.

- **Outpatient physician visits** were defined as days with a record for German Practitioners' Fee Scale within the Statutory Health Insurance Scheme (Einheitlicher Bewertungsmaßstab, EBM) code and assessed as number of physician visits as well as number and percentage of patients visiting a physician in the respective observation period for cases as well as the matched controls.
- **Hospitalizations** were assessed as number and percentage of patients with a hospitalization and duration of respective inpatient stays for cases and the matched controls. The length of an inpatient stay was calculated by using the following formula:
  $$discharge\ date-admission\ date + 1\ day\!\,\!=\!\,\!length\ of\ hospital\ stay\ in\ days$$
- **Outpatient/inpatient services and procedures** were assessed using EBM and German Key of operations and procedures (Operationen- und Prozedurenschlüssel, OPS) codes as number and percentage of women undergoing procedures in the respective observation period for cases and matched controls and were reported separately for inpatient and outpatient care.

### Statistical methods

We performed a descriptive analysis of outcomes of interest, aiming at showing cost and resources reimbursed by the SHI in Germany as they occurred. No adjustment of the data was performed, e.g., zero cost cases were not excluded as it was deemed plausible that costs in this young cohort should be low.

Healthcare costs and resource utilization for cases and controls were assessed by using univariate statistics as appropriate for continuous (mean, standard deviation, minimum, 25^th^ percentile, median, 75^th^ percentile, and maximum) or categorical (percentage) variables. Due to potential outliers in the data and associated skewness, we focused on medians as a measure of central tendency.

Increments were determined as differences between cases and controls. For continuous variables, increments are reported as both mean and median differences. Differences in proportions were determined as differences in percentages between cases and controls.

The reported results for healthcare costs and healthcare resources are unadjusted results as incurred for the SHI for the years 2012–2017 in Germany.

Analyses were performed in collaboration with the Institute for Applied Health Research Berlin (InGef) GmbH.

## Results

### Study population

The 2,972 women with HPV-associated anogenital diseases identified in the timeframe from 2012–2017 in our prior work [5] were matched to 29,720 women without any record for HPV-associated anogenital diseases from the same birth cohort (1:10 matching ratio) during the 3-year observation period.

### Healthcare resource use

**Fig. 1:**
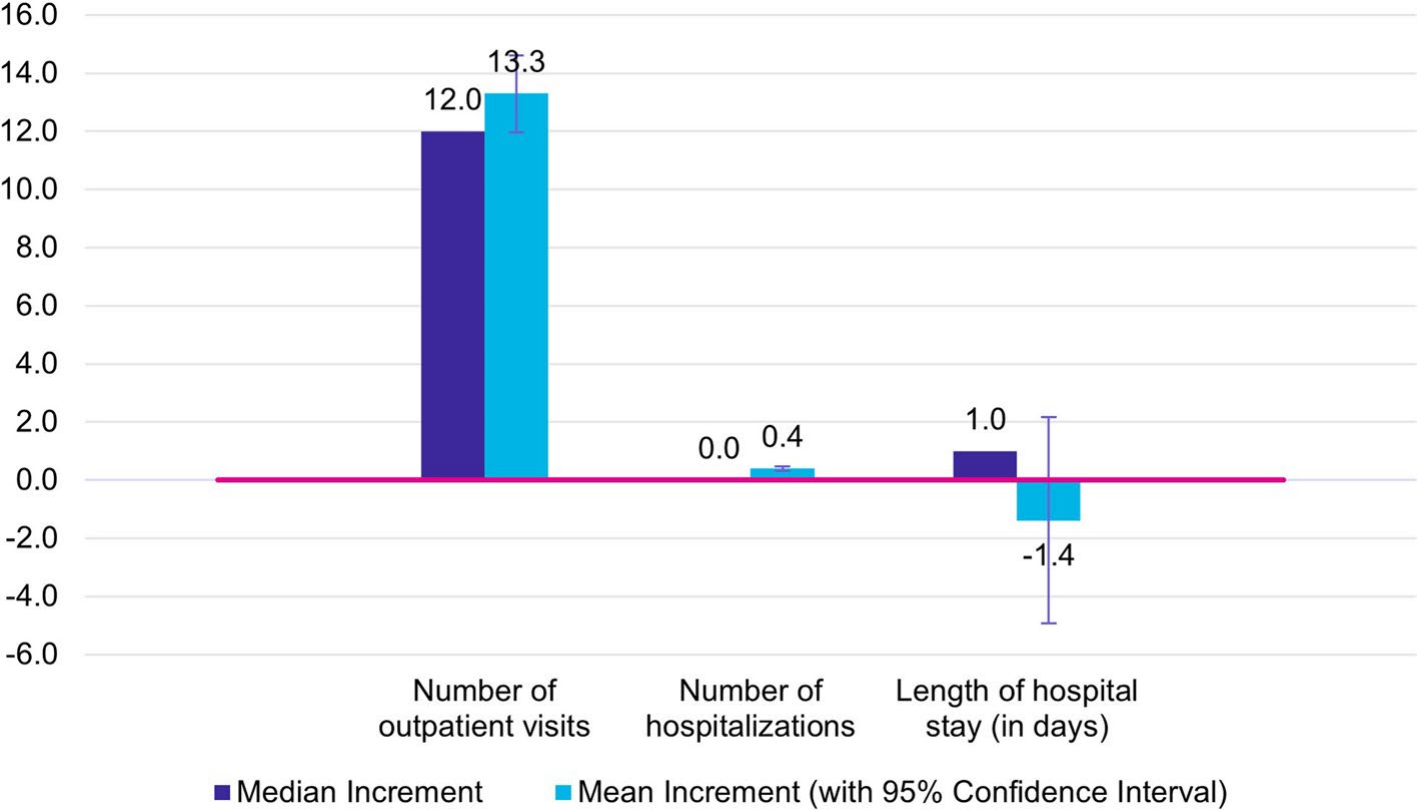
Incremental healthcare resource utilization of cases in relation to controls during 3-year observation period

Median frequency of outpatient physician visits was higher in cases than in controls (45.0 vs. 33.0), leading to 12.0 more visits for women with anogenital diseases in the 3-year observation period (Fig. 1).

Overall, 45.2% of cases and 31.7% of controls had at least one hospitalization within the 3-year observation period (Table 2). If hospitalized, the median length of hospital stay, was 6.0 days in cases and 5.0 days in controls, resulting in a median difference of 1.0 day (Fig. 1).

**Table 2:** All-cause healthcare resource utilization for 23–25-year-old women during 3-year observation period

	**Outpatient visits**^a^		**Hospitalizations**^a^		**Length of hospital stay in days**^b^	
	**Cases**	**Controls**	**Cases**	**Controls**	**Cases**	**Controls**
% of patients with resource utilization	100.0	99.6	45.2	31.7		
Mean	52.4	39.2	1.1	0.7	19.3	20.6
SD	35.6	30.6	2.2	1.6	60.9	69.7
Min	4.0	0.0	0.0	0.0	1.0	1.0
Q1	32.0	21.0	0.0	0.0	3.0	3.0
Median	45.0	33.0	0.0	0.0	6.0	5.0
Q3	64.0	50.0	1.0	1.0	11.0	10.0
Max	1,071.0	1,203.0	51.0	38.0	895.0	946.0

Abbreviations: *SD* Standard deviation, *Min* Minimum, *Q1* 25^th^ percentile, *Q3* 75^th^ percentile, *Max* Maximum

^a^Descriptive statistics are based on 2,972 cases and 29,720 controls

^b^Descriptive statistics are based on 1,344 cases and 9,434 controls with at least one hospitalization

Healthcare resource utilization was also stratified by each birth cohort. The stratified results by birth cohort can be found in Additional file 1, Tables 1, 2, 3 and 4.

Looking at any performed procedures by healthcare domain, 26.8% of cases and 14.1% of controls underwent at least one procedure in the outpatient setting and 36.9% of cases and 23.9% of controls underwent at least one procedure in the inpatient setting. The most common outpatient performed procedure was conization of the cervix uteri based on OPS code: 5–671 (4.4% in cases; n < 5 in controls), followed other excision and destruction of diseased tissue of the cervix uteri based on OPS code: 5–672 (3.1% in cases; 0.0% in controls) (Table 3). Overall, the observed top 10 outpatient performed procedures in cases during the 3-year observation period are shown in Table 3 and almost all were related to diagnostic and surgery of anogenital regions. In the inpatient setting, most of the observed top 10 performed procedures were related to pregnancy and labor. Besides these, cervical conization was the most common performed procedure in 4.7% of cases (Table 4).

**Table 3:** Top 10 outpatient procedures in cases performed for any cause during the 3-year observation period

OPS code	Description	Cases		Controls	
		n	%	n	%
At least one procedure		796	26.8	29,585	14.1
5–671.0	Conization of the cervix uteri: conization	132	4.4	< 5	-
5–672.0	Other excision and destruction of diseased tissue of the cervix uteri: excision	91	3.1	12	0.0
1–650.2	Diagnostic colonoscopy: total, with ileoscopy	64	2.2	351	1.2
5–690.0	Therapeutic curettage [abrasio uteri]: without local drug application	46	1.5	114	0.4
1–472.0	Biopsy without incision of the cervix uteri: cervical abrasion	44	1.5	17	0.1
1–672	Diagnostic hysteroscopy	44	1.5	69	0.2
5–702.1	Local excision and destruction of diseased tissue of the vagina and the douglas area: excision of diseased tissue of the vagina	42	1.4	< 5	-
5–895.2a	Radical and extensive excision of diseased tissue on the skin and subcutaneous tissue: with primary wound closure: chest wall and back	36	1.2	325	1.1
1–471.2	Biopsy without incision on the endometrium: Diagnostic fractional curettage	28	0.9	20	0.1
1–650.1	Diagnostic colonoscopy: total up to the caecum	22	0.7	106	0.4
1–661	Diagnostic urethrocystoscopy	22	0.7	196	0.7

Abbreviations: *OPS* Operationen- und Prozedurenschlüssel [Key of operations and procedures]

**Table 4:** Top 10 inpatient procedures in cases performed for any cause during the 3-year observation period

OPS code	Description	Cases		Controls	
		n	%	n	%
At least one procedure		1,097	36.9	7,112	23.9
9–262.0	Postnatal care of the newborn: routine care	223	7.5	1,676	5.6
5–671.0	Conization of the cervix uteri: conization	141	4.7	< 5	-
9–260	Supervise and direct of normal birth	118	4.0	958	3.2
1–208.8	Registration of evoked potentials: otoacoustic emissions	105	3.5	803	2.7
8–910	Epidural injection and infusion for pain management	104	3.5	637	2.1
1–472.0	Biopsy without incision of the cervix uteri: cervical abrasion	95	3.2	6	0.0
9–261	Supervision and management of risk birth	81	2.7	592	2.0
1–650.2	Diagnostic colonoscopy: total, with ileoscopy	77	2.6	298	1.0
5–758.2	Reconstruction of female genital organs after rupture, post-partum [perineal tear]: vagina	60	2.0	412	1.4
9–262.1	Postnatal care of the newborn: special care (high-risk newborn)	56	1.9	388	1.3

Abbreviations: *OPS* Operationen- und Prozedurenschlüssel [Key of operations and procedures]

For both cases and controls the top 10 of prescribed substances were mostly pain medications as well as antibiotics. The most common prescribed substance was ibuprofen (40.7% in cases and 37.2% in controls), followed by metamizole sodium (24.8% in cases and 20.1% in controls), and cefuroxime (22.6% in cases and 17.2% in controls). A list of the top 10 percentages prescribed substances in cases can be found in the Additional file 1, Table 5. Prescribed substances for the treatment of genital warts and anogenital lesions in women with anogenital diseases included podophyllotoxin (4.9% in cases), imiquimod (3.8% in cases), and sinecatechine (green tea) (3.3% in cases).

**Table 5:** Healthcare costs (€) for 23–25-year-old women during 3-year observation period

	**Total costs**		**Outpatient care**		**Inpatient care**		**Pharmaceutical costs**	
	**Cases**	**Controls**	**Cases**	**Controls**	**Cases**	**Controls**	**Cases**	**Controls**
Mean	4,119	3,030	1,714	1,266	1,646	1,220	759	544
SD	8,906	9,256	1,394	1,383	4,970	4,910	5,459	6,732
Min	130	0	76	0	0	0	0	0
Q1	1,089	665	907	584	0	0	44	26
Median	1,883	1,199	1,336	940	0	0	100	70
Q3	4,292	3,086	2,025	1,538	1,762	681	212	165
Max	167,999	688,293	14,182	86,056	107,171	306,853	123,006	680,668

Costs are displayed in Euros (€). Descriptive statistics are based on 2,972 cases and 29,720 controls

Abbreviations: *SD* Standard deviation, *Min* Minimum, *Q1* 25^th^ percentile, *Q3* 75^th^ percentile, *Max* Maximum

### Healthcare costs

**Fig. 2:**
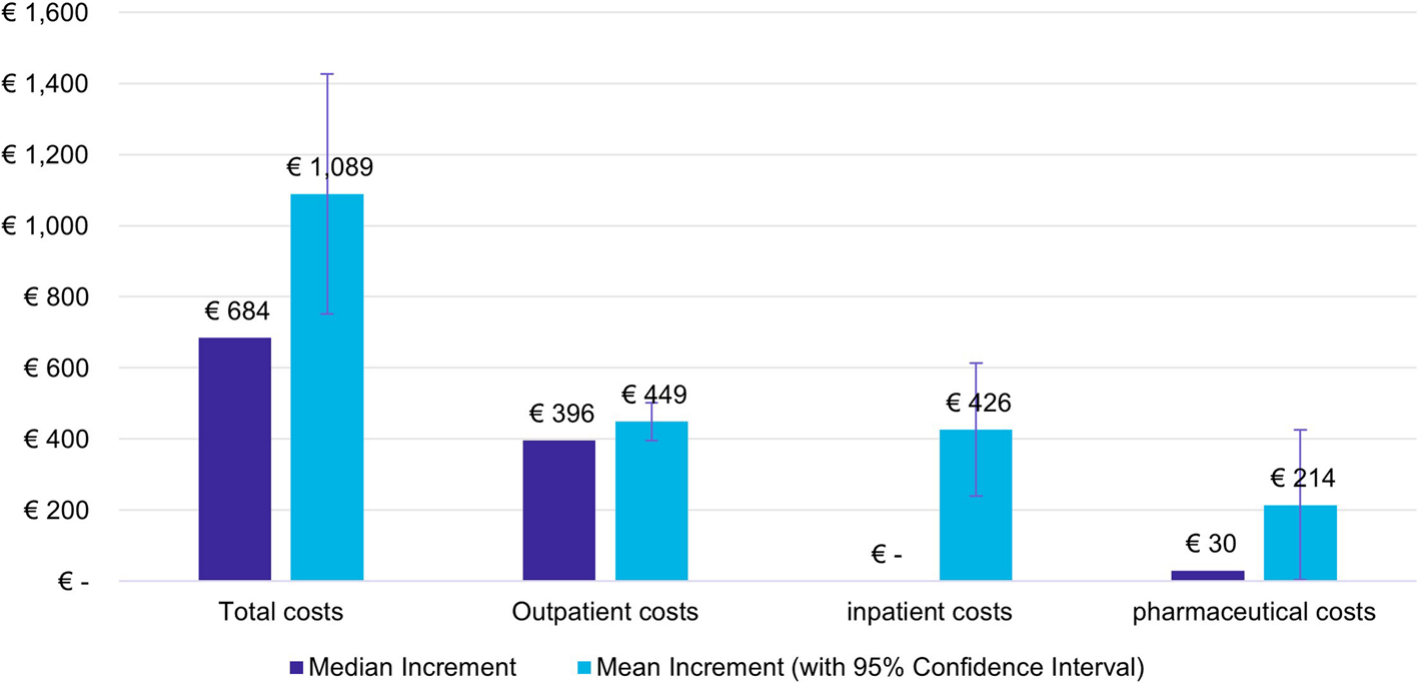
Incremental healthcare costs of cases in relation to controls during 3-year observation period

Overall, women with anogenital diseases had higher median and mean total healthcare costs (consisting of costs for outpatient care, inpatient care, and outpatient pharmaceuticals) than women without anogenital diseases within the 3-year observation period (Table 5). Incremental mean total healthcare costs of €1,089 (95%CI: €752–1,426) (Fig. 2) suggest that HPV-related anogenital diseases were responsible for approximately €3.2 Million more healthcare costs for the identified 2,792 cases in the four birth cohorts within the 3-year observation period in the InGef Research Database.

Higher increments in total healthcare costs in cases were mainly driven by costs for outpatient care. Outpatient and inpatient costs accounted for 41.6% and 40.0% of total healthcare costs in cases. Mean incremental outpatient costs of €449 (95%CI: €396–501) (Fig. 2) suggest that HPV related anogenital diseases increased outpatient healthcare expenditure by €1.3 Million for the identified women in the InGef Research Database born 1989–1992 during the 3-year observation period.

Healthcare costs were also stratified by each birth cohort. The stratified results by birth cohort can be found in Additional file 1, Tables 6, 7, 8 and 9.

## Discussion

The aim of this study was to describe the health economic burden of HPV-associated anogenital diseases in terms of healthcare resource utilization and healthcare costs in a previously identified population of 23–25-year-old women with HPV-associated anogenital diseases [5].

We found that in Germany anogenital diseases in young women were associated with considerable resource utilization and costs during a 3-year observation period, particularly for outpatient care. Considering our 2,972 identified females with anogenital diseases and the mean incremental cost of €1,089, we estimate that HPV-related anogenital diseases were responsible for approximately €3.2 Million costs for the identified women in the InGef Research Database born 1989–1992 within the 3-year observation period. Extrapolating the mean incremental costs to the German population of 23-year old women born 1989–1992 (around 1.9 million women [19]) and assuming 3-year administrative prevalence rate from those same years for HPV-associated anogenital diseases, we estimate that HPV-associated anogenital diseases produced approximately €99.9 Million more costs for the German population of 23–25-year-old women within the three years.

To our knowledge, this was the first study assessing the health economic burden of HPV-associated anogenital diseases in a population of young women from the perspective of the German SHI that covers several anogenital diagnoses associated with HPV. Previous studies have focused on single diagnoses. Hillemanns et al. [6] conducted a multi-center cross-sectional cost-of-illness study surveying gynecologists, dermatologists, and urologists who treated patients aged 14–65 years with genital warts at multiple sites in Germany between February and April 2005. Petry et al. [7] used a study design similar to the design of Hillemanns et al. estimating costs of CIN based on medical records and associated unit costs from EBM codes for a cohort of women with a mean age of 39 years. Hampl et al. [8] estimated the economic burden in women induced by surgical interventions and related diagnostic procedures for grade 2/3 vulvar and vaginal neoplasia using retrospectively collected data from German patient records between 1991 and 2008. None of the mentioned studies is comparable to our study due to differences in analyzed anatomic sites, data sources, study designs, and especially age of the study populations. We intentionally analyzed a consiberably younger cohort of women with anogenital diseases. However, it needs to be considered that disease management in young women may be more restrictive, e.g., for CIN [11], and some anogenital diseases typically occur in older age groups. From our findings no estimates on the healthcare resource utilization and costs of older age groups can be made. This also needs to be considered when looking at resource utilization and prescription rates.

The observed prescriptions indicate that pharmaceutical treatment was applied according to the guideline recommendation for treatment of genital warts and anogenital lesions concerning podophyllotoxin (4.9% in cases), imiquimod (3.8% in cases), and sinecatechine (green tea) (3.3% in cases) [10]. Imiquimod and podophyllotoxin have also been reported as the most frequently self-administered medications for patients with genital warts elsewhere [6]. We also found that more cases than controls received prescriptions for pain medications as well as antibiotics. These higher prescription rates might be related to the procedures performed in cases e.g., cervical conization. However, due to our descriptive study design, it is not possible to identify post-surgical pharmaceutical treatments, so that we can only assume that the higher prescription rates are related to higher procedure rates.

Besides the stated features of this study, the use of claims data to investigate research questions has specific limitations, which needs to be considered when interpreting the results. Claims data is primarily collected for reimbursement purposes, i.e., only data of patients who saw a physician and got a diagnosis record for a respective anogenital disease and caused reimbursement for the health insurance were available in the database. Undiagnosed patients as well as patients who did not receive any treatment that could be reimbursed by the SHI (e.g., over-the-counter drugs) were not captured with this analysis. This might have led to an underestimation of the actual healthcare expenditure.

For the identification of HPV infection and HPV related anogenital diseases, we used ICD-10-GM codes, which is the official classification for the encoding of diagnoses in inpatient and outpatient medical care in Germany since 2000 [20]. In general, clinicians in the outpatient setting must add one of the following specifications to the ICD-10-GM codes: “suspected diagnosis”, “diagnosis ruled out”, “condition after the respective diagnosis”, or “verified diagnosis”. For instance, “suspected” is used, if the physician is not certain about the presence of the coded disease and a confirming laboratory test is still pending. To ensure the accuracy of diagnoses only verified diagnoses in the outpatient and primary and secondary diagnoses in the inpatient setting were used in this study. With this approach, however, we excluded women who might have been suspected to have one of the investigated anogenital diseases or women who had been cured from of the anogenital diseases (e.g., genital warts) and saw their physician for a follow-up visit. Additionally, we decided to only consider very specific ICD-10-GM codes potentially associated with HPV-associated anogenital diseases, but physicians might have used less specific or other codes, e.g., abnormal Pap smear was not considered.

In Germany, cervical cancer screening is Pap-based for women aged 20–34 years [9]. Therefore, most of the documented diagnoses in our analysis were most likely defined cytologically or histologically. Although laboratory data is not linked to the claims database, given the known epidemiology of anogenital precancers and cancers, it is expected that high-risk HPV infections cause almost all cervical cancers and precancers, approximately 90% of high-grade anal, vulvar and vaginal intraepithelial neoplasia and 30, 70 and 90% of vulvar, vaginal and anal cancers, respectively [21].

Our study did not define an index event with follow-up period to track specific disease related healthcare consumption and expenditures but instead assessed the totality of HPV-related diseases costs for diagnostics, examinations, treatments, and follow-up. Due to the definition of the observation period by calendar years rather than a follow-up period after an index event, it is possible that the observation period in our case study population might also include a disease-free period, e.g., before a diagnosis or after complete remission. The decision to use calendar years for the observation period was made to report costs and resource utilization from the administrative perspective of the German SHI system. As some of the included diseases have a longer latency time and are rare in young women, we decided on a 3-year observation period in order to include as many women with anogenital diseases as possible in this young age group. Furthermore, the 3-year oberservation period provides the opportunity to capture prolonged treatment periods. Often the captured diseases require continuous management and can not be treated with one time intervention.

No methods to adjust for cost inflation were used in this study. Because the study period covered only a six year period, it was expected that the inflation rate would have a negligible impact on the cost analyses.

## Conclusion

This study demonstrates that the burden of HPV-associated anogenital disease in 23–25-year-old women in Germany is associated with considerable resource and financial expenditure for the SHI system, mostly driven by outpatient and inpatient care. Our study provides a broader view of healthcare consumption and costs of HPV-associated diseases in young women than has been previously reported for the German SHI. Prevention of HPV-associated diseases in this population would decrease both individual and third-party payer burden.

## Supplementary Information

**Additional file 1:** **Supplementary material Table 1.** Healthcare resource use for 23-25-year-old women during 3-year observation period – birth cohort 1989. **Table 2.** Healthcare resource use for 23-25-year-old women during 3-year observation period – birth cohort 1990. **Table 3**. Healthcare resource use for 23-25-year-old women during 3-year observation period – birth cohort 1991. **Table 4**. Healthcare resource use for 23-25-year-old women during 3-year observation period – birth cohort 1992. **Table 5**. Top 10 outpatient prescribed substances in cases during the 3-year observation period. **Table 6.** Healthcare costs for 23-25-year-old women during 3-year observation period – birth cohort 1989. **Table 7.** Healthcare costs for 23-25-year-old women during 3-year observation period – birth cohort 1990. **Table 8.** Healthcare costs for 23-25-year-old women during 3-year observation period – birth cohort 1991. **Table 9.** Healthcare costs for 23-25-year-old women during 3-year observation period – birth cohort 1992.

## Data Availability

The data used in this study was retrieved from the Institute for Applied Health Research Berlin (InGef) Research Database (www.ingef.de) and cannot be made available in the manuscript, the supplemental files, or in a public repository due to German data protection laws (Bundesdatenschutzgesetz). To facilitate the replication of results, anonymized data used for this study are stored on a secure drive at the Institute for Applied Health Research Berlin (InGef) GmbH. Access to the data used in this study can only be provided to external parties under the conditions of the cooperation contract of this research project and can be assessed upon request, after written approval at InGef GmbH (Tel. + 49 (30) 21 23 36–471; info@ingef.de), if required.

## Abbreviations

AIN: Anal intraepithelial neoplasia
ATC: Anatomical Therapeutic Chemical (Classification System)
BC: Birth cohort
CIN: Cervical intraepithelial neoplasia
DESTATIS: Statistisches Bundesamt [Federal Office of Statistics]
EBM: Einheitlicher Bewertungsmaßstab [Practitioners' Fee Scale within the Statutory Health Insurance Scheme]
HPV: Human Papilloma Virus
ICD-10-GM: International Classification of Disease, 10th Revision, German Modification
InGef: Institut für angewandte Gesundheitsforschung Berlin [Institute for Applied Health Research Berlin]
Max: Maximum
Min: Minimum
OPS: Operationen- und Prozedurenschlüssel [Key of operations and procedures]
OTC: Over-the-counter
Q1: 25^Th^ percentile
Q3: 75^Th^ percentile
SD: Standard deviation
SHI: Statutory Health Insurance
VaIN: Vaginal intraepithelial neoplasia
VIN: Vulvar intraepithelial neoplasia

## References

[1] World Health Organization. Human papillomavirus (HPV) and cervical cancer. Available from: https://www.who.int/news-room/fact-sheets/detail/human-papillomavirus-(hpv)-and-cervical-cancer. Accessed 4 Aug 2022.

[2] Ferris DG, Brown DR, Giuliano AR, Myers E, Joura EA, Garland SM, Kjaer SK, Perez G, Saah A, Luxembourg A (2020). Prevalence, incidence, and natural history of HPV infection in adult women ages 24 to 45 participating in a vaccine trial. Papillomavirus Res (Amsterdam, Netherlands).

[3] De Vuyst H, Clifford GM, Nascimento MC, Madeleine MM, Franceschi S (2009). Prevalence and type distribution of human papillomavirus in carcinoma and intraepithelial neoplasia of the vulva, vagina and anus: a meta-analysis. Int J Cancer.

[4] Ho GY, Bierman R, Beardsley L, Chang CJ, Burk RD (1998). Natural history of cervicovaginal papillomavirus infection in young women. N Engl J Med.

[5] Reuschenbach M, Mihm S, Wölle R, Schneider KM, Jacob C, Braun S, Greiner W, Hampl M (2020). Burden of HPV related anogenital diseases in young women in Germany - an analysis of German statutory health insurance claims data from 2012 to 2017. BMC Infect Dis.

[6] Hillemanns P, Breugelmans JG, Gieseking F, Bénard S, Lamure E, Littlewood KJ, Petry KU (2008). Estimation of the incidence of genital warts and the cost of illness in Germany: A cross-sectional study. BMC Infect Dis.

[7] Petry K, Breugelmans G, Benard S, Lamure E, Littlewood K, Hillemanns P (2008). Cost of screening and treatment of cervical dyskaryosis in Germany. Eur J Gynaecol Oncol.

[8] Hampl M, Huppertz E, Schulz-Holstege O, Kok P, Schmitter S (2011). Economic burden of vulvar and vaginal intraepithelial neoplasia: retrospective cost study at a German dysplasia centre. BMC Infect Dis.

[9] Gemeinsamer Bundesausschuss. Programm zur Früherkennung von Gebärmutterhalskrebs. Available from: https://www.g-ba.de/themen/methodenbewertung/ambulant/frueherkennung-krankheiten/erwachsene/krebsfrueherkennung/gebaermutterhalskrebs-screening/. Accessed 4 Aug 2022.

[10] Gross GE, Werner RN, Becker JC, Brockmeyer NH, Esser S, Hampl M, et al. S2k Leitlinie - HPV-assoziierte Läsionen der äußeren Genitalregion und des Anus – Genitalwarzen und Krebsvorstufen der Vulva, des Penis und der peri- und intraanalen Haut (AWMF-Registernummer: 082-008). Available from: https://www.awmf.org/uploads/tx_szleitlinien/082-008l_S2k_HPV_assoziierte_anogenitale_L%C3%A4sionen_2017-11-verlaengert.pdf. Accessed 4 Aug 2022. 10.1111/ddg.13441_g 29418090

[11] Leitlinienprogramm Onkologie (Deutsche Krebsgesellschaft, Deutsche Krebshilfe, AWMF). Prävention des Zervixkarzinoms, Langversion 1.1, 2020, AWMF Registernummer: 015/027OL. Available from: https://www.awmf.org/uploads/tx_szleitlinien/015-027OLl_Praevention_Zervixkarzinom_2020-03-verlaengert.pdf. Accessed 4 Aug 2022.

[12] Diagnosis, Therapy, and Follow-Up Care of Vulvar Cancer and its Precursors. National Guideline of the German Society of Gynecology and Obstetrics (S2k-Level, AWMF Registry No. 015/059, August 2015). Available from: https://www.pathologie.de/?eID=downloadtool&uid=1407. Accessed 4 Aug 2022.

[13] Bundesministerium für Gesundheit. Kennzahlen der Gesetzlichen Krankenversicherung 2008 bis 2021. Kennzahlen und Faustformeln. Available from: https://www.bundesgesundheitsministerium.de/fileadmin/Dateien/3_Downloads/Statistiken/GKV/Kennzahlen_Daten/KF2021Bund_Juli_2021.pdf. Accessed 4 Aug 2022.

[14] Statistisches Bundesamt. Ergebnisse der Bevölkerungsfortschreibung auf Grundlage des Zensus 2011. Available from: https://www.destatis.de/DE/Themen/Gesellschaft-Umwelt/Bevoelkerung/Bevoelkerungsstand/Tabellen/liste-zensus-geschlecht-staatsangehoerigkeit.html. Accessed 4 Aug 2022.

[15] GKV-Spitzenverband. Die gesetzlichen Krankenkassen. Anzahl der Krankenkassen im Zeitablauf – Konzentrationsprozess durch Fusionen (Angaben am Stichtag 1.1.). Available from: https://www.gkv-spitzenverband.de/krankenversicherung/kv_grundprinzipien/alle_gesetzlichen_krankenkassen/alle_gesetzlichen_krankenkassen.jsp. Accessed 4 Aug 2022.

[16] Statistisches Bundesamt. Ergebnisse der Bevölkerungsfortschreibung auf Grundlage des Zensus 2011. Available from: https://www.destatis.de/DE/Themen/Gesellschaft-Umwelt/Bevoelkerung/Bevoelkerungsstand/_inhalt.html. Accessed 4 Aug 2022.

[17] Andersohn F, Walker J (2016). Characteristics and external validity of the German Health Risk Institute (HRI) Database. Pharmacoepidemiol Drug Saf.

[18] Ludwig M, Enders D, Basedow F, Walker J, Jacob J (2022). Sampling strategy, characteristics and representativeness of the InGef research database. Public Health.

[19] Statistisches Bundesamt. Fortschreibung des Bevölkerungsstandes Deutschland - Bevölkerung: Deutschland, Stichtag, Altersjahre, Nationalität/Geschlecht/Familienstand. Available from: https://www-genesis.destatis.de/genesis/online?operation=abruftabelleBearbeiten&levelindex=2&levelid=1629810259830&auswahloperation=abruftabelleAuspraegungAuswaehlen&auswahlverzeichnis=ordnungsstruktur&auswahlziel=werteabruf&code=12411-0006&auswahltext=&nummer=5&variable=5&name=GES&werteabruf=Werteabruf#abreadcrumb. Accessed 4 Aug 2022.

[20] Bundesinstitut für Arzneimittel und Medizinprodukte. ICD-10-GM - Versionsverlauf und Bekanntmachungen. Available from: https://www.dimdi.de/dynamic/de/klassifikationen/icd/icd-10-gm/historie/versionsverlauf/. Accessed 4 Aug 2022.

[21] Hartwig S, Baldauf J-J, Dominiak-Felden G, Simondon F, Alemany L, de Sanjosé S, Castellsagué X (2015). Estimation of the epidemiological burden of HPV-related anogenital cancers, precancerous lesions, and genital warts in women and men in Europe: Potential additional benefit of a nine-valent second generation HPV vaccine compared to first generation HPV vaccines. Papillomavirus Res.

