# Supplementary material for: Healthcare resource utilization and costs in 23–25-year-old women with human papillomavirus (HPV) associated anogenital diseases in Germany – a retrospective analysis of statutory health insurance claims data
Source: BMC Health Serv Res. 2022 Aug 5;22:1002. doi: 10.1186/s12913-022-08397-1 (PMC9356499; doi:10.1186/s12913-022-08397-1)
Supplement: Supplementary file 1 — Additional file 1: Supplementary material Table 1. Healthcare resource use for 23-25-year-old women during 3-year observation period – birth cohort 1989. Table 2. Healthcare resource use for 23-25-year-old women during 3-year observation period – birth cohort 1990. Table 3. Healthcare resource use for 23-25-year-old women during 3-year observation period – birth cohort 1991. Table 4. Healthcare resource use for 23-25-year-old women during 3-year observation period – birth cohort 1992. Table 5. Top 10 outpatient prescribed substances in cases during the 3-year observation period. Table 6. Healthcare costs for 23-25-year-old women during 3-year observation period – birth cohort 1989. Table 7. Healthcare costs for 23-25-year-old women during 3-year observation period – birth cohort 1990. Table 8. Healthcare costs for 23-25-year-old women during 3-year observation period – birth cohort 1991. Table 9. Healthcare costs for 23-25-year-old women during 3-year observation period – birth cohort 1992. [file 12913_2022_8397_MOESM1_ESM.docx]

**Supplementary material**

**Table 1** Healthcare resource use for 23-25-year-old women during 3-year observation period – birth cohort 1989

|  | **Cases** | **Controls** | **Increment** |
| --- | --- | --- | --- |
| Individuals in cohort |  |  |  |
| N (%) | 846 (100.0) | 8,460(100.0) |  |
| Outpatient visits |  |  |  |
| N (%) | 846 (100.0) | 8,414 (99.5) |  |
| Sum | 41,640.0 | 329,675.0 |  |
| Mean (all individuals in cohort) | 49.2 | 39.0 | 10.3 |
| SD | 28.4 | 34.8 |  |
| Min | 4.0 | 0.0 |  |
| Q1 | 31.0 | 21.0 |  |
| Median | 44.0 | 32.0 | 12.0 |
| Q3 | 59.0 | 50.0 |  |
| Max | 244.0 | 1,115.0 |  |
| Hospitalizations |  |  |  |
| N (%) | 383 (45.3) | 2,724 (32.2) |  |
| Sum | 890.0 | 5,559.0 |  |
| Mean (all individuals in cohort) | 1.1 | 0.7 | 0.4 |
| SD | 2.0 | 1.5 |  |
| Min | 0.0 | 0.0 |  |
| Q1 | 0.0 | 0.0 |  |
| Median | 0.0 | 0.0 | 0.0 |
| Q3 | 1.0 | 1.0 |  |
| Max | 19.0 | 29.0 |  |
| Length of hospital stay |  |  |  |
| N (%) | 383 (45.3) | 2,724 (32.2) |  |
| Sum | 6,703.0 | 49,339.0 |  |
| Mean (individuals with hospital stay) | 17.5 | 18.1 | -0.6 |
| SD | 62.4 | 56.8 |  |
| Min | 1.0 | 1.0 |  |
| Q1 | 3.0 | 3.0 |  |
| Median | 5.0 | 5.0 | 0.0 |
| Q3 | 10.0 | 10.0 |  |
| Max | 856.0 | 905.0 |  |

Abbreviations: *SD*: standard deviation, *Min*: minimum, *Q1*: 25^th^ percentile, *Q3*: 75^th^ percentile, *Max*: maximum.

**Table 2** Healthcare resource use for 23-25-year-old women during 3-year observation period – birth cohort 1990

|  | **Cases** | **Controls** | **Increment** |
| --- | --- | --- | --- |
| Individuals in cohort |  |  |  |
| N (%) | 811 (100.0) | 8,110 (100.0) |  |
| Outpatient visits |  |  |  |
| N (%) | 811 (100.0) | 8,080 (99,6 |  |
| Sum | 43,121.0 | 313,571.0 |  |
| Mean (all individuals in cohort) | 53.2 | 38.7 | 14.5 |
| SD | 33.2 | 27.5 |  |
| Min | 7.0 | 0.0 |  |
| Q1 | 32.5 | 21.0 |  |
| Median | 46.0 | 32.0 | 14.0 |
| Q3 | 65.0 | 49.0 |  |
| Max | 382.0 | 524.0 |  |
| Hospitalizations |  |  |  |
| N (%) | 363 (44.8) | 2,565 (31.6) |  |
| Sum | 907.0 | 5,407.0 |  |
| Mean (all individuals in cohort) | 1.1 | 0.7 | 0.5 |
| SD | 2.6 | 1.6 |  |
| Min | 0.0 | 0.0 |  |
| Q1 | 0.0 | 0.0 |  |
| Median | 0.0 | 0.0 | 0.0 |
| Q3 | 1.0 | 1.0 |  |
| Max | 51.0 | 38.0 |  |
| Length of hospital stay |  |  |  |
| N (%) | 363 (44.8) | 2,565 (31.6) |  |
| Sum | 5,559.0 | 52,020.0 |  |
| Mean (individuals with hospital stay) | 15.3 | 20.3 | -5.0 |
| SD | 39.0 | 69.0 |  |
| Min | 1.0 | 1.0 |  |
| Q1 | 3.0 | 3.0 |  |
| Median | 6.0 | 5.0 | 1.0 |
| Q3 | 11.0 | 10.0 |  |
| Max | 446.0 | 942.0 |  |

Abbreviations: *SD*: standard deviation, *Min*: minimum, *Q1*: 25^th^ percentile, *Q3*: 75^th^ percentile, *Max*: maximum.

**Table 3** Healthcare resource use for 23-25-year-old women during 3-year observation period – birth cohort 1991

|  | **Cases** | **Controls** | **Increment** |
| --- | --- | --- | --- |
| Individuals in cohort |  |  |  |
| N (%) | 650 (100.0) | 6,500 (100.0) |  |
| Outpatient visits |  |  |  |
| N (%) | 650 (100.0) | 6,473 (99.6) |  |
| Sum | 35,442.0 | 258,017.0 |  |
| Mean (all individuals in cohort) | 54.5 | 39.7 | 14.8 |
| SD | 49.3 | 28.9 |  |
| Min | 6.0 | 0.0 |  |
| Q1 | 34.0 | 21.0 |  |
| Median | 46.0 | 33.0 | 13.0 |
| Q3 | 65.0 | 51.0 |  |
| Max | 1,071.0 | 771.0 |  |
| Hospitalizations |  |  |  |
| N (%) | 301 (46.3) | 2,054 (31.6) |  |
| Sum | 707.0 | 4,669.0 |  |
| Mean (all individuals in cohort) | 1.1 | 0.7 | 0.4 |
| SD | 1.9 | 1.8 |  |
| Min | 0.0 | 0.0 |  |
| Q1 | 0.0 | 0.0 |  |
| Median | 0.0 | 0.0 | 0.0 |
| Q3 | 1.0 | 1.0 |  |
| Max | 18.0 | 34.0 |  |
| Length of hospital stay |  |  |  |
| N (%) | 301 (46.3) | 2,054 (31.6) |  |
| Sum | 7,724.0 | 49,643.0 |  |
| Mean (individuals with hospital stay) | 25.7 | 24.2 | 1.5 |
| SD | 81.1 | 81.6 |  |
| Min | 1.0 | 1.0 |  |
| Q1 | 3.0 | 3.0 |  |
| Median | 6.0 | 5.0 | 1.0 |
| Q3 | 11.0 | 10.0 |  |
| Max | 895.0 | 946.0 |  |

Abbreviations: *SD*: standard deviation, *Min*: minimum, *Q1*: 25^th^ percentile, *Q3*: 75^th^ percentile, *Max*: maximum.

**Table 4** Healthcare resource use for 23-25-year-old women during 3-year observation period – birth cohort 1992

|  | **Cases** | **Controls** | **Increment** |
| --- | --- | --- | --- |
| Individuals in cohort |  |  |  |
| N (%) | 665 (100.0) | 6,650 (100.0) |  |
| Outpatient visits |  |  |  |
| N (%) | 665 (100.0) | 6,618 (99.5) |  |
| Sum | 35,639.0 | 262,288.0 |  |
| Mean (all individuals in cohort) | 53.6 | 39.4 | 14.2 |
| SD | 30.0 | 30.0 |  |
| Min | 9.0 | 0.0 |  |
| Q1 | 33.0 | 21.0 |  |
| Median | 47.0 | 33.0 | 14.0 |
| Q3 | 65.0 | 50.0 |  |
| Max | 223.0 | 1,203.0 |  |
| Hospitalizations |  |  |  |
| N (%) | 297 (44.7) | 2,091 (31.4) |  |
| Sum | 691.0 | 4,522.0 |  |
| Mean (all individuals in cohort) | 1.0 | 0.7 | 0.4 |
| SD | 2.0 | 1.6 |  |
| Min | 0.0 | 0.0 |  |
| Q1 | 0.0 | 0.0 |  |
| Median | 0.0 | 0.0 | 0.0 |
| Q3 | 1.0 | 1.0 |  |
| Max | 18.0 | 23.0 |  |
| Length of hospital stay |  |  |  |
| N (%) | 297 (44.7) | 2,091 (31.4) |  |
| Sum | 5,886.0 | 43,529.0 |  |
| Mean (individuals with hospital stay) | 19.8 | 20.8 | -1.0 |
| SD | 56.8 | 72.8 |  |
| Min | 1.0 | 1.0 |  |
| Q1 | 3.0 | 2.0 |  |
| Median | 5.0 | 5.0 | 0.0 |
| Q3 | 12.0 | 9.0 |  |
| Max | 500.0 | 852.0 |  |

Abbreviations: *SD*: standard deviation, *Min*: minimum, *Q1*: 25^th^ percentile, *Q3*: 75^th^ percentile, *Max*: maximum.

**Table 5** Top 10 outpatient prescribed substances in cases during the 3-year observation period

| **ATC code** | **Description** | **Cases** | | **Controls** | |
| --- | --- | --- | --- | --- | --- |
|  |  | n | % | n | % |
| At least one prescription | | 2,864 | 96.4 | 27,527 | 92.6 |
| M01AE01 | Ibuprofen | 1,209 | 40.7 | 11,046 | 37.2 |
| N02BB02 | Metamizole sodium | 738 | 24.8 | 5,971 | 20.1 |
| J01DC02 | Cefuroxime | 673 | 22.6 | 5,106 | 17.2 |
| J01CA04 | Amoxicillin | 668 | 22.5 | 5,811 | 19.6 |
| A02BC02 | Pantoprazole | 571 | 19.2 | 4,777 | 16.1 |
| J01MA02 | Ciprofloxacin | 528 | 17.8 | 4,055 | 13.6 |
| A03FA01 | Metoclopramide | 524 | 17.6 | 4,103 | 13.8 |
| J01XX01 | Fosfomycin | 503 | 16.9 | 3,389 | 11.4 |
| J01FA10 | Azithromycin | 475 | 16.0 | 4,237 | 14.3 |
| J01AA02 | Doxycycline | 416 | 14.0 | 3,008 | 10.1 |

Abbreviations: *ATC*: Anatomical Therapeutic Chemical (Classification System)

**Table 6** Healthcare costs for 23-25-year-old women during 3-year observation period – birth cohort 1989

|  | **Cases** | **Controls** | **Increment** |
| --- | --- | --- | --- |
| Individuals in cohort |  |  |  |
| N (%) | 846 (100.0) | 8,460 (100.0) |  |
| Total costs |  |  |  |
| Sum | 3,334,052 | 25,300,591 |  |
| Mean (all individuals in cohort) | 3,941 | 2,991 | 950 |
| SD | 9,766 | 10,786 |  |
| Min | 172 | 0 |  |
| Q1 | 1,025 | 640 |  |
| Median | 1,736 | 1,159 | 577 |
| Q3 | 3,835 | 3,062 |  |
| Max | 167,999 | 688,293 |  |
| Inpatient care |  |  |  |
| Sum | 1,203,629 | 10,121,906 |  |
| Mean (all individuals in cohort) | 1,423 | 1,196 | 226 |
| SD | 3,926 | 4,651 |  |
| Min | 0 | 0 |  |
| Q1 | 0 | 0 |  |
| Median | 0 | 0 | 0 |
| Q3 | 1,713 | 699 |  |
| Max | 55,684 | 177,713 |  |
| Outpatient care |  |  |  |
| Sum | 1,301,665 | 10,350,807 |  |
| Mean (all individuals in cohort) | 1,539 | 1,223 | 315 |
| SD | 1,147 | 1,458 |  |
| Min | 76 | 0 |  |
| Q1 | 843 | 557 |  |
| Median | 1,219 | 907 | 312 |
| Q3 | 1,823 | 1,497 |  |
| Max | 11,027 | 83,152 |  |
| Pharmaceuticals |  |  |  |
| Sum | 828,758 | 4,827,879 |  |
| Mean (all individuals in cohort) | 980 | 571 | 409 |
| SD | 7,169 | 8,983 |  |
| Min | 0 | 0 |  |
| Q1 | 42 | 27 |  |
| Median | 98 | 71 | 27 |
| Q3 | 199 | 170 |  |
| Max | 123,006 | 680,668 |  |

Costs are displayed in Euros (€).

Abbreviations: *SD*: standard deviation, *Min*: minimum, *Q1*: 25^th^ percentile, *Q3*: 75^th^ percentile, *Max*: maximum.

**Table 7** Healthcare costs for 23-25-year-old women during 3-year observation period – birth cohort 1990

|  | **Cases** | **Controls** | **Increment** |
| --- | --- | --- | --- |
| Individuals in cohort |  |  |  |
| N (%) | 811 (100.0) | 8,110 (100.0) |  |
| Total costs |  |  |  |
| Sum | 3,596,871 | 23,176,634 |  |
| Mean (all individuals in cohort) | 4,435 | 2,858 | 1,577 |
| SD | 10,320 | 8,023 |  |
| Min | 237 | 0 |  |
| Q1 | 1,048 | 651 |  |
| Median | 1,936 | 1,178 | 758 |
| Q3 | 4,383 | 2,969 |  |
| Max | 164,844 | 386,163 |  |
| Inpatient care |  |  |  |
| Sum | 1,511,128 | 9,541,963 |  |
| Mean (all individuals in cohort) | 1,863 | 1,177 | 687 |
| SD | 6,356 | 5,444 |  |
| Min | 0 | 0 |  |
| Q1 | 0 | 0 |  |
| Median | 0 | 0 | 0 |
| Q3 | 1,836 | 681 |  |
| Max | 107,171 | 306,853 |  |
| Outpatient care |  |  |  |
| Sum | 1,430,458 | 10,110,955 |  |
| Mean (all individuals in cohort) | 1,764 | 1,247 | 517 |
| SD | 1,548 | 1,251 |  |
| Min | 230 | 0 |  |
| Q1 | 890 | 576 |  |
| Median | 1,362 | 927 | 435 |
| Q3 | 2,122 | 1,505 |  |
| Max | 14,182 | 21,620 |  |
| Pharmaceuticals |  |  |  |
| Sum | 655,284 | 3,523,716 |  |
| Mean (all individuals in cohort) | 808 | 434 | 374 |
| SD | 5,466 | 3,617 |  |
| Min | 0 | 0 |  |
| Q1 | 45 | 26 |  |
| Median | 102 | 71 | 31 |
| Q3 | 212 | 160 |  |
| Max | 78,288 | 169,138 |  |

Costs are displayed in Euros (€).

Abbreviations: *SD*: standard deviation, *Min*: minimum, *Q1*: 25^th^ percentile, *Q3*: 75^th^ percentile, *Max*: maximum.

**Table 8** Healthcare costs for 23-25-year-old women during 3-year observation period – birth cohort 1991

|  | **Cases** | **Controls** | **Increment** |
| --- | --- | --- | --- |
| Individuals in cohort |  |  |  |
| N (%) | 650 (100.0) | 6,500 (100.0) |  |
| Total costs |  |  |  |
| Sum | 2,700,321 | 20,467,025 |  |
| Mean (all individuals in cohort) | 4,154 | 3,149 | 1,006 |
| SD | 7,758 | 7,941 |  |
| Min | 130 | 0 |  |
| Q1 | 1,128 | 680 |  |
| Median | 2,009 | 1,227 | 782 |
| Q3 | 4,542 | 3,164 |  |
| Max | 71,281 | 218,116 |  |
| Inpatient care |  |  |  |
| Sum | 1,072,932 | 8,597,347 |  |
| Mean (all individuals in cohort) | 1,651 | 1,323 | 328 |
| SD | 4,761 | 5,063 |  |
| Min | 0 | 0 |  |
| Q1 | 0 | 0 |  |
| Median | 0 | 0 | 0 |
| Q3 | 1,819 | 699 |  |
| Max | 59,107 | 177,706 |  |
| Outpatient care |  |  |  |
| Sum | 1,136,501 | 8,433,245 |  |
| Mean (all individuals in cohort) | 1,748 | 1,297 | 451 |
| SD | 1,368 | 1,585 |  |
| Min | 130 | 0 |  |
| Q1 | 943 | 592 |  |
| Median | 1,366 | 963 | 403 |
| Q3 | 2,094 | 1,576 |  |
| Max | 13,336 | 86,056 |  |
| Pharmaceuticals |  |  |  |
| Sum | 490,888 | 3,436,433 |  |
| Mean (all individuals in cohort) | 755 | 529 | 227 |
| SD | 5,113 | 4,635 |  |
| Min | 0 | 0 |  |
| Q1 | 43 | 25 |  |
| Median | 101 | 70 | 30 |
| Q3 | 233 | 164 |  |
| Max | 62,595 | 169,429 |  |

Costs are displayed in Euros (€).

Abbreviations: *SD*: standard deviation, *Min*: minimum, *Q1*: 25^th^ percentile, *Q3*: 75^th^ percentile, *Max*: maximum.

**Table 9** Healthcare costs for 23-25-year-old women during 3-year observation period – birth cohort 1992

|  | **Cases** | **Controls** | **Increment** |
| --- | --- | --- | --- |
| Individuals in cohort |  |  |  |
| N (%) | 665 (100.0) | 6,650 (100.0) |  |
| Total costs |  |  |  |
| Sum | 2,609,783 | 21,106,669 |  |
| Mean (all individuals in cohort) | 3,924 | 3,174 | 751 |
| SD | 6,678 | 9,733 |  |
| Min | 287 | 0 |  |
| Q1 | 1,151 | 701 |  |
| Median | 2,028 | 1,263 | 765 |
| Q3 | 4,466 | 3,151 |  |
| Max | 118,025 | 358,940 |  |
| Inpatient care |  |  |  |
| Sum | 1,103,350 | 7,995,177 |  |
| Mean (all individuals in cohort) | 1,659 | 1,202 | 457 |
| SD | 4,396 | 4,363 |  |
| Min | 0 | 0 |  |
| Q1 | 0 | 0 |  |
| Median | 0 | 0 | 0 |
| Q3 | 1,727 | 611 |  |
| Max | 58,069 | 158,351 |  |
| Outpatient care |  |  |  |
| Sum | 1,226,404 | 8,720,205 |  |
| Mean (all individuals in cohort) | 1,844 | 1,311 | 533 |
| SD | 1,485 | 1,214 |  |
| Min | 207 | 0 |  |
| Q1 | 957 | 615 |  |
| Median | 1,433 | 980 | 453 |
| Q3 | 2,162 | 1,602 |  |
| Max | 11,163 | 22,432 |  |
| Pharmaceuticals |  |  |  |
| Sum | 280,029 | 4,391,286 |  |
| Mean (all individuals in cohort) | 421 | 660 | -239 |
| SD | 2,400 | 7,931 |  |
| Min | 0 | 0 |  |
| Q1 | 47 | 26 |  |
| Median | 104 | 70 | 35 |
| Q3 | 220 | 168 |  |
| Max | 51,328 | 351,879 |  |

Costs are displayed in Euros (€).

Abbreviations: *SD*: standard deviation, *Min*: minimum, *Q1*: 25^th^ percentile, *Q3*: 75^th^ percentile, *Max*: maximum.
